# Baseline Double-Balloon Enteroscopy Findings and Long-Term Outcomes in Pediatric Crohn’s Disease

**DOI:** 10.7759/cureus.103632

**Published:** 2026-02-14

**Authors:** Marcio Roberto Facanali, Carolina Bortolozzo Graciolli Facanali, Marcelo Rodrigues Borba, Carlos Walter Sobrado, Adriana V Safatle-Ribeiro

**Affiliations:** 1 Gastroenterology, Hospital das Clínicas da Faculdade de Medicina da Universidade de São Paulo, São Paulo, BRA

**Keywords:** crohn disease, device-assisted enteroscopy, double-balloon enteroscopy, enteroscopy, harvey-bradshaw index, simple endoscopic score for crohn’s disease, small intestine

## Abstract

Background

Pediatric Crohn’s disease is frequently characterized by extensive small-bowel involvement and a dynamic disease course. Double-balloon enteroscopy (DBE) allows direct evaluation of proximal small-bowel lesions that may not be detected by conventional upper gastrointestinal endoscopy and ileocolonoscopy; however, the long-term clinical implications of baseline DBE findings remain poorly defined.

Methods

We conducted a retrospective, single-center longitudinal cohort study including pediatric patients initially diagnosed with Crohn’s disease who underwent baseline DBE. Clinical and endoscopic disease activity were assessed using the Harvey-Bradshaw Index (HBI) and the Simple Endoscopic Score for Crohn’s Disease (SES-CD), respectively, at baseline and after approximately 10 years of follow-up. Patients with diagnostic reclassification during follow-up were excluded from remission analyses but analyzed descriptively.

Results

Twenty patients underwent baseline DBE; three (15%) were subsequently reclassified to alternative diagnoses. Among the remaining 17 (85%) patients with a final diagnosis of Crohn’s disease, both clinical and endoscopic disease activity substantially improved over long-term follow-up. Baseline DBE identified ulcerative jejunal disease in a subset of patients. At follow-up, patients with ulcerative lesions identified on baseline DBE tended to exhibit lower rates of clinical and endoscopic remission compared with those without proximal ulceration. These subgroup findings were exploratory in nature.

Conclusions

In pediatric patients initially diagnosed with Crohn’s disease, baseline DBE findings were associated with distinct long-term clinical and endoscopic trajectories. Additionally, the observed rate of diagnostic reclassification underscores the importance of longitudinal reassessment in this population. DBE may provide complementary phenotypic information beyond standard endoscopic evaluation in selected pediatric patients.

## Introduction

Crohn’s disease in pediatric patients is characterized by marked clinical heterogeneity and a dynamic disease course, frequently involving extensive segments of the gastrointestinal tract at diagnosis. Compared with adults, children often present with more widespread disease, including small-bowel involvement, which has important implications for prognosis and long-term management [1]. Accurate initial phenotyping is therefore essential, as early disease characteristics may influence therapeutic strategies, long-term outcomes, and the need for surgical intervention [2,3].

Assessment of the small bowel represents a central challenge in the evaluation of pediatric Crohn’s disease. Conventional ileocolonoscopy, while fundamental for diagnosis, is limited to the colon and terminal ileum and may underestimate proximal small-bowel involvement. Cross-sectional imaging techniques provide indirect information on inflammatory activity but do not allow direct mucosal assessment or tissue sampling. Capsule endoscopy has significantly expanded visualization of the small bowel and demonstrated high sensitivity for detecting proximal lesions, however, its use may be limited by the risk of capsule retention, particularly in patients with known or suspected stricturing disease, as well as by the inability to obtain histological samples [4,5].

Device-assisted enteroscopy has emerged as an important complementary modality for the evaluation of the small bowel. Double-balloon enteroscopy (DBE), first described as a method for achieving total enteroscopy, allows direct visualization of the entire small bowel, targeted biopsies, and therapeutic interventions when required [6]. In pediatric populations, DBE has been shown to be feasible and safe, providing additional diagnostic and therapeutic information in selected patients with suspected or confirmed Crohn’s disease, particularly when proximal small-bowel involvement is suspected or when other diagnostic modalities are inconclusive [7].

Beyond its diagnostic role, DBE may contribute to a more refined characterization of disease phenotype at presentation. In pediatric Crohn’s disease, initial phenotypic features are increasingly recognized as relevant to disease behavior and long-term outcomes, in line with contemporary management strategies that emphasizeindividualized assessment and treat-to-target approaches [8,9]. Current consensus guidelines underscore the importance of individualized assessment and close monitoring in patients with features suggestive of a more complex disease course, including extensive or proximal small-bowel involvement [2,3].

In parallel, it is well recognized that a subset of pediatric patients initially diagnosed with Crohn’s disease may later undergo diagnostic reclassification to other immune-mediated or monogenic inflammatory conditions, particularly in very early-onset disease. Disorders affecting the interleukin-10 pathway represent a paradigmatic example, as they may closely mimic Crohn’s disease at presentation while following a distinct clinical course over time [10]. This diagnostic overlap further highlights the importance of comprehensive initial evaluation and longitudinal reassessment in pediatric patients with suspected inflammatory bowel disease.

In this scenario, the present study aimed to evaluate the long-term clinical and endoscopic outcomes of pediatric patients initially diagnosed with Crohn’s disease who underwent baseline DBE. By comparing disease activity at baseline and after approximately 10 years of follow-up, we sought to explore whether ulcerative proximal small-bowel findings identified on DBE are associated with distinct long-term disease trajectories.

## Materials and methods

Study design and population

This was a retrospective, single-center longitudinal cohort study conducted at a quaternary referral hospital, Hospital das Clínicas da Faculdade de Medicina da Universidade de São Paulo, located in São Paulo, Brazil, including pediatric patients initially diagnosed with Crohn’s disease who underwent DBE between 2009 and 2020. During this period, a total of 20 pediatric patients with suspected or confirmed Crohn’s disease were evaluated at our institution, and all 20 underwent DBE as part of their diagnostic work-up.

All consecutive pediatric patients who underwent DBE for suspected or confirmed Crohn’s disease during the study period and who had available clinical and endoscopic data at baseline and at long-term follow-up were eligible for inclusion. No eligible patients who underwent DBE during the study period were excluded due to missing baseline or follow-up data.

Patients were followed longitudinally for approximately 10 years. During follow-up, diagnostic reclassification was recorded when applicable. Patients who underwent diagnostic reclassification were excluded from remission analyses but were retained for descriptive evaluation.

Double-balloon enteroscopy

DBE was performed using the double-balloon technique, as originally described, with the aim of evaluating the proximal small bowel beyond the reach of conventional upper gastrointestinal endoscopy and ileocolonoscopy. The indication for DBE included suspected proximal small-bowel involvement, inconclusive findings on other diagnostic modalities, or persistent symptoms despite standard evaluation.

Enteroscopy findings were reviewed retrospectively. For the purposes of this study, ulcerative proximal small-bowel disease was defined by the presence of ulcerative lesions in the jejunum or duodenum identified on baseline DBE. Non-ulcerative findings included normal mucosa, erythema, nodularity, or cicatricial changes without active ulceration.

Clinical and endoscopic assessment

Clinical disease activity was assessed using the Harvey-Bradshaw Index (HBI) [11]. Clinical remission was defined as an HBI score <5. Endoscopic disease activity was assessed using the Simple Endoscopic Score for Crohn’s Disease (SES-CD) [12], derived from ileocolonoscopic evaluations. Endoscopic remission was defined as an SES-CD score ≤2.

Baseline clinical and endoscopic assessments were performed in close temporal proximity to the DBE procedure. In routine clinical practice, ileocolonoscopy and clinical assessment were performed approximately 30 days after DBE, and this interval was considered acceptable to define baseline status for the purposes of this study.

Clinical and endoscopic assessments were performed at two predefined time points: at baseline and at long-term follow-up. No missing data were present for HBI or SES-CD at these time points among patients included in the remission analyses.

Long-term follow-up and outcomes

The primary outcome of interest was long-term clinical and endoscopic activity at follow-up, assessed by HBI and SES-CD, respectively. The secondary outcome included diagnostic reclassification to other inflammatory or immune-mediated conditions.

In patients who underwent surgical intervention during follow-up, endoscopic activity was assessed descriptively based on the available postoperative evaluations of the remaining intestinal segments.

Statistical analysis

Given the exploratory nature of the study and the limited sample size, analyses were primarily descriptive. Continuous variables are presented as means with standard deviations or medians with ranges, as appropriate. Categorical variables are presented as counts and percentages. Paired comparisons between baseline and long-term follow-up were performed using non-parametric tests, whereas subgroup analyses according to baseline DBE findings were exploratory and descriptive only. All analyses were performed using IBM SPSS Statistics for Windows, version 22.0 (IBM Corp., Armonk, New York, United States).

Ethical considerations

The study was approved by the Brazilian National Research Ethics Committee through Plataforma Brasil (approval number: CAAE 35880720.3.0000.0068). Given the retrospective nature of the study, informed consent requirements were waived in accordance with local regulations. All data were anonymized prior to analysis.

## Results

A total of 20 (100%) pediatric patients with an initial diagnosis of Crohn’s disease underwent baseline DBE and were included in the cohort. During long-term follow-up, three (15%) patients underwent diagnostic reclassification, including one (5%) interleukin-10-related monogenic disease, one (5%) juvenile idiopathic arthritis-associated intestinal disease, and one (5%) ulcerative colitis. The remaining 17 (85%) patients retained a final diagnosis of Crohn’s disease and were included in the primary analyses of long-term Crohn’s disease outcomes. Baseline demographic and disease characteristics of the cohort are summarized in Table 1.

**Table 1:** Baseline demographic and disease characteristics (N = 17)

Variable	Value
Age, years	
Mean ± SD	11.4 ± 5.4
Median (min; max)	12 (3; 20)
Sex, n (%)	
Male	11 (64.7)
Female	6 (35.3)
Disease behaviour, n (%)	
Non-penetrating / non-stenosing	9 (52.9)
Stenosing	4 (23.5)
Penetrating	4 (23.5)
Perianal disease, n (%)	
No	11 (64.7)
Yes	6 (35.3)

**Figure 1:**
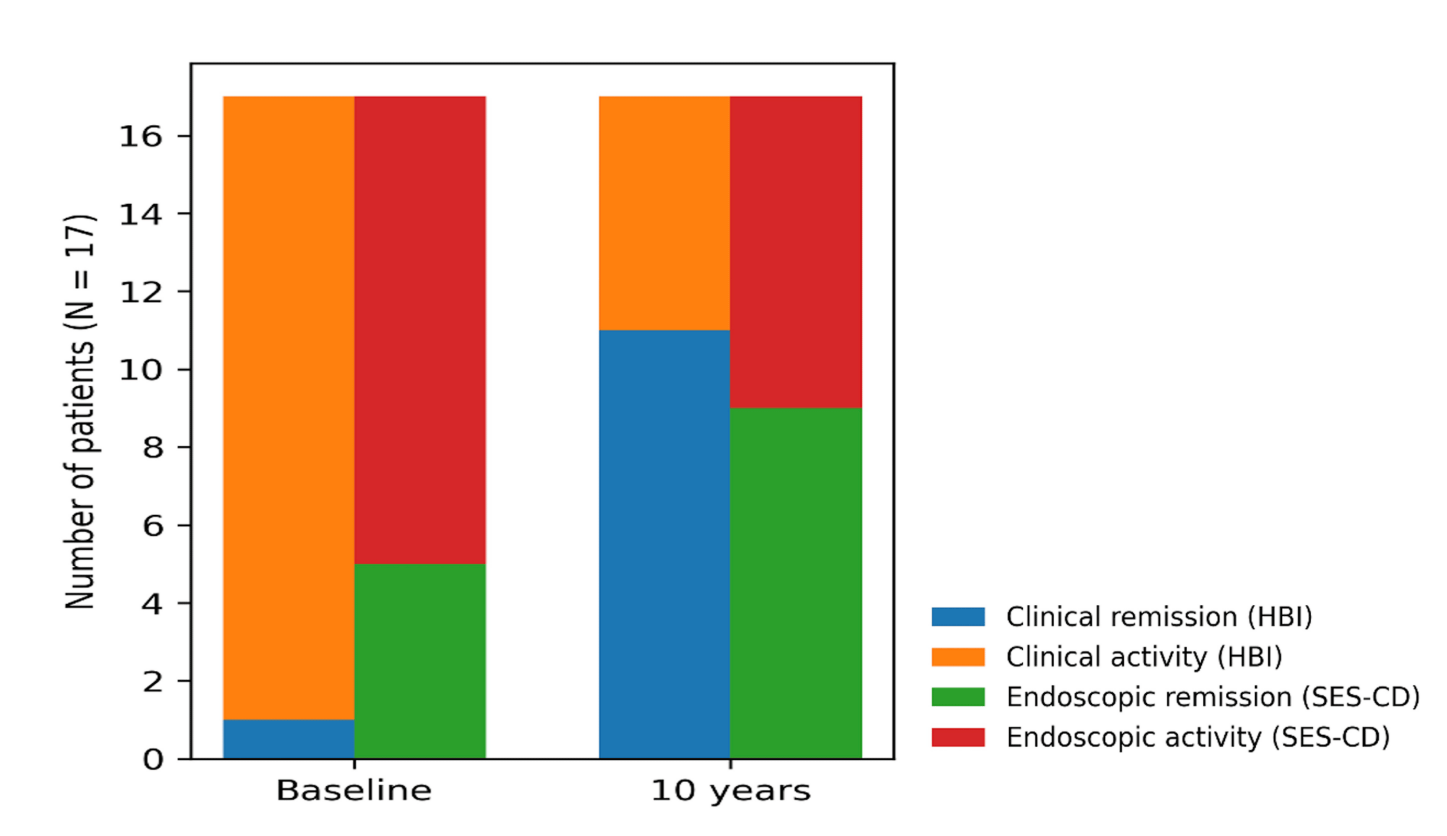
Clinical and endoscopic status at baseline and long-term follow-up HBI: Harvey-Bradshaw Index; SES-CD: Simple Endoscopic Score for Crohn’s Disease

All patients underwent standardized clinical and endoscopic assessment at two predefined time points: at baseline, in close temporal proximity to DBE, and at long-term follow-up approximately 10 years later. Among 17 (85%) patients with a final diagnosis of Crohn’s disease, clinical disease activity significantly decreased over time, with a marked reduction in HBI scores between baseline and long-term follow-up (p < 0.001) (Figure 1).

Similarly, endoscopic disease activity improved over time, as reflected by a significant reduction in SES-CD scores between baseline and follow-up (p = 0.002). Longitudinal changes in clinical and endoscopic outcomes, including treatment patterns, clinical activity, and endoscopic severity, are detailed in Table 2.

**Table 2:** Longitudinal clinical and endoscopic outcomes Paired Wilcoxon signed-rank test was used for longitudinal comparisons of continuous variables. Test statistics are reported as Z values (two-sided). HBI: Harvey-Bradshaw Index; SES-CD: Simple Endoscopic Score for Crohn’s Disease

Variable	Baseline	10 years	Test statistic (Z)	p-value
Treatment, n (%)				
No medication	1 (5.9)	3 (17.6)	-	-
Immunosuppressor	10 (58.8)	3 (17.6)	-	-
Biologic	2 (11.8)	4 (23.5)	-	-
Immunosuppressor + biologic	4 (23.5)	3 (17.6)	-	-
Surgery	0 (0)	2 (11.8)	-	--
Surgery + biologic	0 (0)	2 (11.8)	-	-
Clinical status (HBI), n (%)				
Remission	1 (5.9)	11 (64.7)	-	-
Mild	5 (29.4)	2 (11.8)	-	-
Moderate	7 (41.2)	3 (17.6)	-	-
Severe	4 (23.5)	1 (5.9)	-	-
HBI score				
Mean ± SD	11.1 ± 5.3	4.3 ± 5.3	3.57	0.001
Median (min; max) n=17	10 (3; 22)	1 (0; 19)		
Endoscopic activity (SES-CD), n (%)				
Remission	5 (29.4)	9 (52.9)	-	-
Mild	0 (0)	3 (17.6)	-	--
Moderate	4 (23.5)	4 (23.5)	-	-
Severe	8 (47.1)	1 (5.9)	-	-
SES-CD score				
Mean ± SD	15.4 ± 12.9	4.2 ± 6.1	3.04	0.012
Median (min; max) n=17	15 (0; 43)	0 (0; 22)		

**Figure 2:**
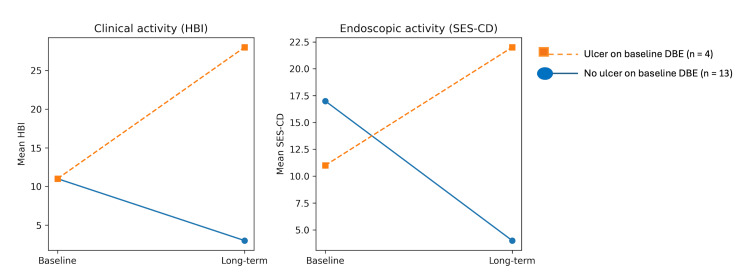
Clinical and endoscopic activity at follow-up according to baseline enteroscopic ulceration DBE: double-balloon enteroscopy; HBI: Harvey-Bradshaw Index; SES-CD: Simple Endoscopic Score for Crohn’s Disease

At baseline, DBE identified ulcerative jejunal disease in a subset of patients, whereas the remaining patients showed non-ulcerative proximal small-bowel findings. At long-term follow-up, patients with ulcerative lesions identified on baseline DBE tended to present lower rates of clinical remission compared with those without jejunal ulceration. These findings are presented descriptively and should be interpreted as exploratory, given the limited sample size (Figure 2).

Diagnostic reclassification occurred in three patients (15%) during follow-up. These cases were excluded from remission analyses and analyzed descriptively. All reclassified patients initially presented with clinical and endoscopic features suggestive of Crohn’s disease and underwent DBE as part of their baseline evaluation, highlighting the phenotypic overlap between Crohn’s disease and other immune-mediated or monogenic inflammatory conditions in pediatric patients.

## Discussion

In this long-term real-world cohort of pediatric patients initially diagnosed with Crohn’s disease, we observed a marked and sustained improvement in both clinical and endoscopic activity over approximately 10 years of follow-up. Importantly, baseline evaluation with DBE provided clinically meaningful information beyond standard ileocolonoscopy, while a non-negligible proportion of patients underwent diagnostic reclassification over time, underscoring the dynamic nature of pediatric inflammatory bowel disease phenotyping.

In this cohort, baseline ulcerative involvement of the proximal small bowel was associated with a distinct long-term disease trajectory. Patients presenting with jejunal ulceration at baseline tended to maintain higher levels of clinical and endoscopic activity over time, whereas those without proximal ulceration generally evolved toward sustained clinical and endoscopic improvement. Previous studies have shown that proximal small-bowel involvement, particularly jejunal disease, is associated with a more severe and complex phenotype of Crohn’s disease, especially in pediatric populations [2,3]. Although exploratory, these findings suggest that ulcerative proximal small-bowel involvement identified at baseline may reflect disease heterogeneity rather than a transient inflammatory manifestation. In this context, DBE may contribute to phenotypic characterization and hypothesis generation regarding disease complexity in pediatric Crohn’s disease.

The significant reduction in clinical activity, as measured by the HBI, together with parallel improvement in endoscopic severity assessed by the SES-CD, aligns with contemporary treat-to-target strategies emphasizing sustained symptom control and mucosal healing as long-term goals in Crohn’s disease management [8,9]. Although pediatric data with extended follow-up remain limited, our findings are consistent with reports suggesting that early disease control can translate into durable clinical benefit, even in patients with initially extensive or proximal disease involvement.

Moreover, endoscopic outcomes were assessed using ileocolonoscopic indices (SES-CD), which may not fully capture proximal small-bowel disease activity. Therefore, DBE findings at baseline should be interpreted as complementary phenotypic information rather than as a comprehensive longitudinal assessment of proximal small-bowel inflammation.

Finally, although long-term clinical and endoscopic improvement was observed in this cohort, treatment strategies evolved substantially over the follow-up period, including escalation to biologic therapy and surgical intervention, as summarized in Table 2. Given the retrospective design and limited sample size, we did not aim to attribute long-term outcomes to specific therapeutic regimens. Accordingly, the observed long-term trajectories should be interpreted as reflecting real-world disease evolution under contemporary management rather than the effect of any single intervention.

A key aspect of this study is the focus on proximal small bowel assessment. Conventional ileocolonoscopy may underestimate disease extent in pediatric Crohn’s disease, particularly when jejunal involvement is present. While small bowel capsule endoscopy has improved mucosal visualization, its inability to obtain tissue samples or perform therapeutic interventions, along with the risk of capsule retention in established disease, limits its applicability in certain clinical scenarios [4,5]. In contrast, DBE allows direct visualization and biopsy of proximal lesions and may help identify a subgroup of patients with more complex disease biology. Although our analyses were exploratory, patients with active jejunal disease on baseline DBE tended to have lower rates of long-term clinical remission, suggesting that proximal inflammatory burden may carry prognostic implications.

Beyond disease activity, diagnostic stability emerged as an important finding. Of patients initially diagnosed with Crohn’s disease, 15% were later reclassified to alternative conditions, including monogenic disease related to interleukin-10 signaling, juvenile idiopathic arthritis-associated intestinal involvement, and ulcerative colitis. This observation is consistent with prior reports highlighting the phenotypic overlap between Crohn’s disease and other immune-mediated or monogenic disorders in pediatric populations, particularly in early-onset or atypical cases [10]. Our findings reinforce the concept that pediatric inflammatory bowel disease should be viewed as an evolving diagnosis, requiring periodic reassessment as new clinical, endoscopic, histologic, or genetic information becomes available.

Taken together, these results support a complementary role for DBE in selected pediatric patients with suspected extensive or proximal disease involvement. Rather than serving solely as a diagnostic tool, DBE may contribute to phenotypic characterization and hypothesis generation by identifying patients with a higher inflammatory burden and atypical disease distribution.

This study has several limitations. First, the retrospective single-center design and the small sample size limit statistical power and preclude robust subgroup analyses; therefore, comparisons according to baseline DBE findings should be interpreted as exploratory and hypothesis-generating. Second, DBE was performed based on clinical indication rather than systematically in all newly diagnosed pediatric Crohn’s disease patients, which introduces potential selection bias and limits the generalizability of our findings to the broader pediatric Crohn’s disease population. Third, treatment strategies evolved substantially over the prolonged follow-up period, including escalation to biologic therapy and surgical intervention, reflecting real-world practice. These treatment differences may have influenced long-term remission outcomes, and no formal adjustment for treatment exposure was performed. Finally, the long duration of follow-up increases the risk of residual confounding related to changes in clinical practice patterns over time.

## Conclusions

In pediatric patients initially diagnosed with Crohn’s disease, baseline DBE findings were associated with distinct long-term clinical and endoscopic trajectories, reinforcing the relevance of deep small-bowel assessment at initial phenotyping. Importantly, the observed rate of diagnostic reclassification highlights the dynamic nature of pediatric inflammatory bowel disease and underscores the need for structured longitudinal reassessment over time. In this context, DBE may contribute complementary phenotypic information beyond that obtained with standard endoscopic evaluation, supporting a more refined characterization of disease behavior and heterogeneity in selected pediatric patients.
